# Prediction accuracy of available early warning systems for air pollution across WHO South-East Asia region: a systematic review

**DOI:** 10.3389/fpubh.2026.1945362

**Published:** 2026-09-30

**Authors:** Charu Mahawar, Sivakumar Sasidharan, Vikram Singh, Manoj Kumar, Neeraj Parmar, Sanghamitra Pati, Sumit Aggarwal

**Affiliations:** 1 Indian Council of Medical Research, ICMR Headquarters, New Delhi, India; 2 National Institute for Research in Environmental Health, Indian Council of Medical Research, Bhopal, India

**Keywords:** air pollution, deep learning, early warning system, India, public health

## Abstract

**Background:**

Ambient air pollution is a major environmental health risk across developing nations in the WHO South-East Asia Region (SEARO), contributing substantially to premature mortality and the global disease burden. While Air Quality Early Warning Systems (AQEWS) enable proactive public health interventions, their regional predictive accuracy remains uncharacterized. Despite their increasing importance, the predictive performance and methodological quality of AQEWS across SEARO have not been systematically evaluated. To address this gap, this study systematically evaluated the accuracy of available AQEWS across the region.

**Methods:**

This systematic review (PROSPERO: CRD420251275901) searched major databases from inception to February 10, 2026, for observational modeling studies reporting quantitative AQEWS performance in SEARO. Reviewers extracted data and assessed bias using PROBAST+AI. Primary outcomes were RMSE, R^2^, categorical accuracy, and forecast bias.

**Results:**

Fourteen studies (India, Bangladesh, Thailand) from 839 records were synthesized; heterogeneity disallowed meta-analysis. Hybrid deep learning architectures consistently yielded the lowest RMSE (0.84–4.61 μg/m^3^) and highest explained variance (*R*^2^ up to 0.998) across the studies, particularly for PM_2.5_ forecasting; however, evidence was heterogeneous and did not establish consistent superiority across pollutants, forecast horizons, validation settings, or operational AQEWS. Machine learning achieved up to 98.34% categorical accuracy. Chemical transport models demonstrated significant bias reduction (~70%) exclusively when integrated with satellite data assimilation. Most studies showed a high risk of bias due to inadequate external validation and algorithmic overfitting.

**Conclusion:**

While hybrid and deep learning models demonstrated superior forecasting accuracy over traditional methods, widespread policy implementation across SEARO was hindered by poor methodological validation. This review provides a regional synthesis of AQEWS forecasting performance and identifies key methodological limitations that currently constrain their reliability for public health decision-making. Standardized reporting and rigorous external validation are urgently required to ensure these systems reliably inform regional public health emergency responses.

**Systematic Review Registration:**

identifier: CRD 420251275901.

## Introduction

Ambient air pollution is established as a leading environmental determinant of the global disease burden, with approximately 99% of the global population residing in areas where air quality fails to meet World Health Organization (WHO) guidelines (1). Despite WHO's 2021 tightened annual mean limits for fine (PM_2.5_, 5 μg/m^3^) and coarse (PM_10_, 15 μg/m^3^) particulate matter, most WHO South-East Asia Region (SEARO) countries exceeded these thresholds several-fold. Globally, 36% of the population remains exposed to PM_2.5_ levels exceeding the 35 μg/m^3^ interim target (2). Consequently, ambient and household air pollution combined cause 6.7 million premature deaths annually. Ambient exposure alone ranks as the fifth-leading global mortality risk factor, driving 4.2 million deaths and 103.1 million disability-adjusted life-years (DALYs). This burden disproportionately impacts low- and middle-income countries (LMICs), which account for 89% of these fatalities. East and South Asia historically represent 59% of PM_2.5_-attributable mortality, exacerbated by extreme population-weighted mean exposures in nations like Bangladesh (89.4 μg/m^3^) and India (74.3 μg/m^3^). In India alone, continuous exposure reduces potential life expectancy by up to 9 years for 40% of the population (3).

The health impacts of this exposure follow distinct demographic and epidemiological vectors. Although air pollution exerts critical impacts on early childhood health, its mortality burden is overwhelmingly characterized by chronic non-communicable diseases, which account for 95% of attributable deaths in adults over 60. Specifically, 68% of ambient air pollution-related premature deaths stem from ischemic heart disease and stroke, 14% from chronic obstructive pulmonary disease, 14% from acute lower respiratory infections, and 4% from lung cancers (1, 3). This distinct epidemiological profile is governed by the pathophysiology of fine particulate matter. Among key pollutants, PM_2.5_ exhibits a significantly stronger association with global mortality than coarse PM_10_ (4). Pathophysiologically, PM_2.5_ and ultrafine particles penetrate lung alveoli into the bloodstream, inducing systemic inflammation, oxidative stress, and endothelial dysfunction more aggressively than PM_10_ (5–7). The consequences are particularly severe in high-exposure corridors (3). Ongoing reliance on coal, transportation emissions, and unchecked urbanization exacerbate these vulnerabilities (8). Furthermore, demographic counter-effects, population growth and aging, routinely offset local healthcare improvements, while greenhouse gas emissions fuel a climate change feedback loop requiring integrated monitoring (3).

Because non-linear integrated exposure-response (IER) risk curves flatten at higher exposure distributions, minor reductions in ambient concentrations within highly polluted environments yield only modest reductions in disease burden unless baseline PM_2.5_ levels drop markedly and sustainably. Therefore, relying solely on long-term mitigation is insufficient to protect public health in the immediate term. This creates an urgent operational necessity for high-fidelity Air Quality Early Warning Systems (AQEWS) to enable real-time risk stratification and prompt emergency responses, communicated to the public via the Air Quality Index (AQI) (9). AQEWS enable proactive public health interventions such as localized emission rationing, school closures, and vulnerable population advisories, before pollution events reach peak toxicity (10). Aligning with this urgent regional necessity, the UN's “Early Warnings for All” initiative targets universal early warning coverage by 2027 (11, 12). However, to achieve this mandate, SEARO's forecasting infrastructure must evolve. Historically, SEARO's forecasting relied on deterministic chemical transport models and physics-based simulations, such as India's System of Air Quality and Weather Forecasting and Research (SAFAR) (13, 14). While mechanistically sound, these resource-intensive frameworks exhibit significant forecast bias due to non-linear spatiotemporal heterogeneity and incomplete regional emission inventories. Consequently, forecasting is shifting toward advanced stochastic modeling and machine learning techniques like Artificial Neural Networks (15–18), Support Vector Machines (19–21), Random Forests (22, 23), and deep learning architectures such as long short-term memory networks (24–28). Integrated with Internet of Things (IoT) sensor arrays, these hybrid frameworks excel at capturing complex temporal dynamics and seasonal emission fluctuations (29).

Despite these advancements, earlier reviews mainly looked at air pollution forecasting algorithms or machine-learning applications in general, and did not specifically focus on forecasting-based AQEWS in the WHO-SEARO; therefore, questions regarding model validity, implementation readiness, and methodological quality remain unanswered. Furthermore, the available evidence remains fragmented across pollutants, forecasting horizons, modeling approaches, performance metrics, and validation strategies, making it difficult to determine the comparative predictive accuracy and methodological reliability of AQEWS across the region. The operational translation of forecasting performance into reliable public health warnings also remains challenging because of heterogeneous monitoring infrastructure, limited data availability, geographic variability, inadequate external validation, and potential model overfitting and poor generalisability across settings.

The main objective of this review was to systematically identify and critically appraise forecasting-based AQEWS developed or evaluated within WHO SEARO countries. Secondary objectives were to compare forecasting methodologies, summarize predictive performance, evaluate methodological quality using PROBAST+AI, identify implementation gaps, and provide recommendations for future operational AQEWS development. By synthesizing this evidence, the review aims to establish the operational readiness of these systems and inform reliable regional public health decision-making.

## Methods

### Search strategy and selection criteria

This systematic review was conducted in accordance with the Preferred Reporting Items for Systematic Reviews and Meta-Analyses (PRISMA) guidelines (30) and was registered with the International Prospective Register of Systematic Reviews (PROSPERO: CRD420251275901). We formulated our primary research question using the Population, Comparison, Outcomes, and Study design (PCOS) framework to evaluate the predictive performance of AQEWS models within the WHO South-East Asia Region (SEARO).

A comprehensive systematic search strategy was deployed to identify relevant literature from database inception until February 10, 2026. Two independent researchers (C.M. and S.S.) searched five electronic databases: MEDLINE (via PubMed), Web of Science, Scopus, Embase, and Google Scholar. This was supplemented by manual backward citation checking of the eligible studies. The search strategy utilized a combination of controlled vocabulary (e.g., MeSH and Emtree terms) and free-text terms including “Air Pollution,” “PM_2.5_,” “PM_10_,” “Model,” “Framework,” “Early Warning System,” and “Systematic Review,” which were adapted with field-specific commands and Boolean operators for each respective database. Detailed search strategy specific for the databases were presented in the Supplementary Table S1. Only publications written in the English language were included in this review.

### PCOS framework and operational definitions

To improve the transparency, reproducibility, and consistency of study selection, the eligibility criteria were prospectively structured using the PCOS (Population, comparator, outcome, study design) framework, which is appropriate for systematic reviews of environmental forecasting and modeling research where intervention–comparator structures are not universally applicable. The framework was operationalised before screening to ensure consistent eligibility decisions across heterogeneous forecasting methodologies.

**Population (P):** studies conducted on evaluating ambient air quality forecasting or forecasting-based Air Quality Early Warning Systems (AQEWS) in the WHO South-East Asia Region (SEARO) Member States were considered for inclusion.

**Comparator (C):** prediction accuracy between the available Air Quality Early Warning Systems (AQEWS) capable of generating prospective predictions of ambient air pollution or Air Quality Index (AQI).

**Outcome (O):** eligible studies had to provide quantitative measures of forecasting performance using recognized validation metrics like RMSE, MAE, MSE, R^2^, correlation coefficient, bias statistics, forecast accuracy, sensitivity, specificity, or equivalent performance indicators.

**Study design (S):** original peer-reviewed studies evaluating forecasting models or operational AQEWS using observational, modeling, retrospective validation, or prospective forecasting designs were eligible. Review articles, conference abstracts, editorials, commentaries, protocols, case reports, gray literature, and studies without original forecasting model evaluation were excluded.

To ensure consistent application of the eligibility criteria, all screening decisions were based on the predefined PCOS framework and accompanying operational definitions. Studies meeting all four PCOS domains were included, whereas studies failing to satisfy one or more predefined criteria were excluded.

For the purpose of this review, forecasting-based AQEWS were operationally defined as systems that provide advance prediction of future pollution episodes before their occurrence, thereby enabling timely public health preparedness and mitigation measures. Studies focusing solely on air-quality monitoring, exposure assessment, pollutant mapping, source apportionment, emission inventories, retrospective analyses, health-effect assessment, or spatial interpolation without a forecasting component were excluded. All potentially eligible studies were reassessed against the predefined eligibility criteria, with particular attention to whether the reported forecasting or early-warning framework provided information relevant to air-quality or pollution-related early warning. Studies were retained when their forecasting outputs contributed to air-quality, pollution, or related environmental early-warning objectives within the scope defined for this review.

### Study selection and data extraction

All retrieved records were exported to the Rayyan systematic review platform, where duplicates were removed based on similarities in article titles, authors, and publication years. Following deduplication, two reviewers (C.M. and S.S.) independently screened the titles and abstracts of the remaining records. Studies failing to meet the predefined selection criteria were excluded. Subsequently, the full texts of all potentially eligible articles were independently evaluated by the same two reviewers. Any disagreements regarding study selection were resolved through discussion or, when necessary, by consulting with senior experts (V.S., M.K., N.P., S.A., and S.P.). A flow diagram detailing the study selection process is presented in Figure 1.

**Figure 1:**
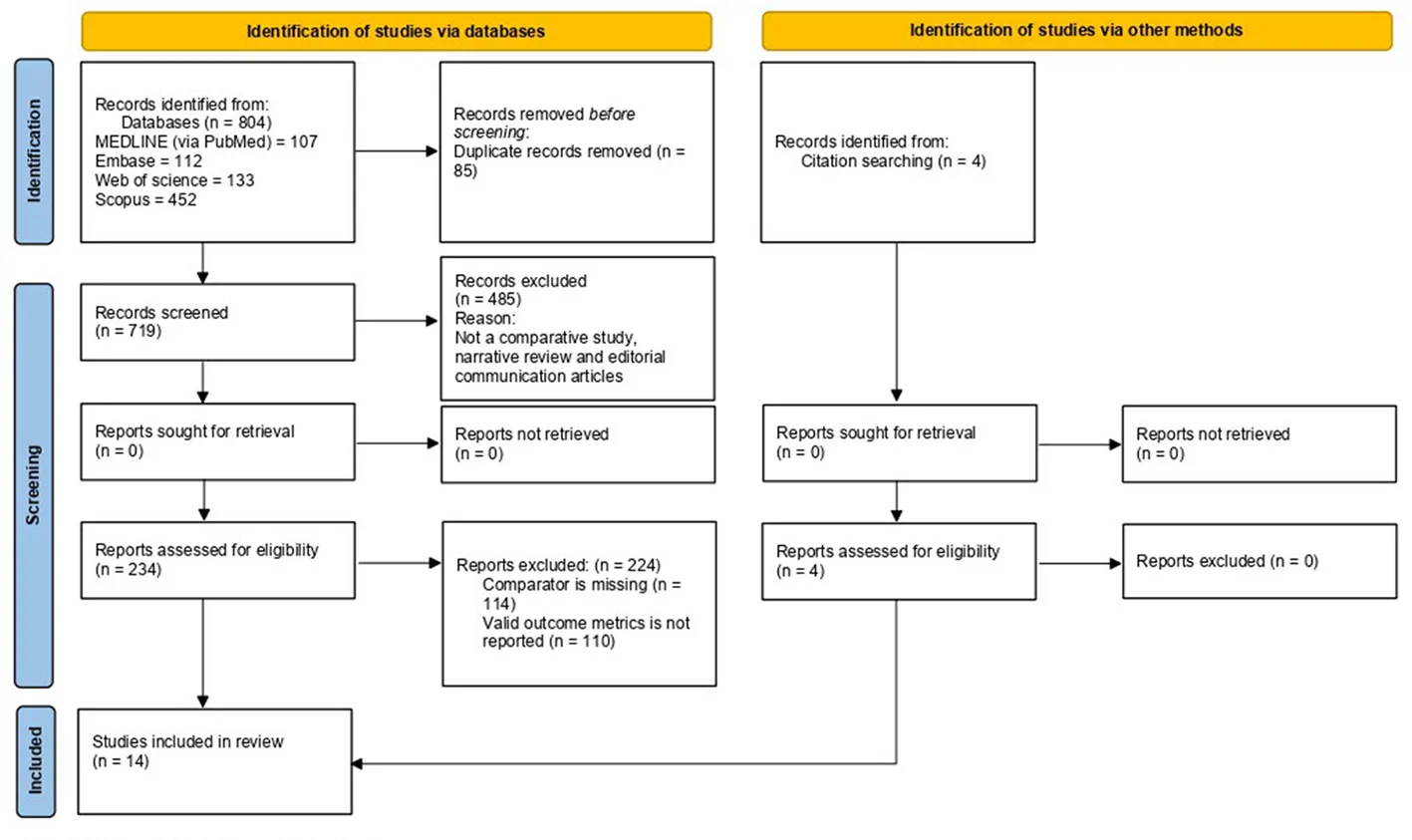
PRISMA flow chart of study selection.

A standardized data extraction form was developed in advance and pilot-tested on five eligible studies. Two reviewers (C.M. and S.S.) independently extracted data from the final included studies. Regular cross-checks were conducted during this process to ensure consistency, and any disagreements were resolved through discussion or consultation with the senior reviewers. The extracted descriptive characteristics for each AQEWS study included the author and publication year, study location, study duration or sample size, system type, studied pollutants, evaluated models or algorithms, validation methods, declared funding sources, and risk-of-bias information (Table 1 and Supplementary Table S2). Furthermore, we extracted specific metric-based performance evaluations from individual studies, capturing data on pollutant concentration, bias (including mean bias and normalized mean bias), Root Mean Square Error (RMSE), Mean Absolute Error (MAE), Mean Squared Error (MSE), R^2^, categorical accuracy, and the Pearson correlation coefficient (Table 2). Variables not reported by the original studies were recorded as “not reported,” and no missing data were imputed.

**Table 1:** Descriptive characteristics of included early warning forecast studies.

S. no	References	Location	Study duration/sample	System type	Pollutants studied	Models/algorithms	Funding
1	Oanh et al. (44)	Chiang Mai, Thailand	2001–2008	Haze alert EWS	PM_10_	M1: Synoptic + Regression,	Nil
						M2: Persistence baseline	
2	Jebamalar et al. (32)	Chennai, India	2017–2019	Public health forecasting EWS	PM_2.5_	M1: Hybrid (LightGBM+DT),	Nil
						M2: LR,	
						M3: Lasso,	
						M4: SVR,	
						M5: NN,	
						M6: DT,	
						M7: RF,	
						M8: XGBoost	
3	Ghude et al. (33)	Delhi & NCR, India	(1) 1–20 Nov 2017 hindcast; (2) 15 Oct 2018–28 Jan 2019 operational	National AQEWS (CTM + satellite assimilation)	PM_2.5_	M1: WRF-Chem + 3DVAR assimilation,	National Monsoon Mission Project, Ministry of Earth Sciences (MoES), India
						M2: WRF-Chem (emissions only)	
4	Jena et al. (34)	Delhi, India	2019–2020	Operational AQEWS	PM_2.5_	M1: WRF-Chem after assimilation,	National Monsoon Mission Project, Ministry of Earth Sciences (MoES), India
						M2: WRF-Chem before assimilation	
5	Gulati et al. (35)	India (Nationwide CPCB)	2021 (1257 stations)	ANN-based Early Warning System	PM_2.5_	M1: LM-ANN,	Nil
						M2: BR-ANN,	
						M3: SCG-ANN,	
						M4: MLR	
6	Kalita et al. (36)	Western India	21–24 Jan 2022 dust event	Operational AQEWS	PM_10_, PM_2.5_	M1: WRF-Chem + GOCART + 3DVAR,	NASA Health and Air Quality Program
						M2: WRF-Chem (WOA)	
7	Saiohai et al. (45)	Bangkok, Thailand	2015–2020 hourly data	Forecasting-based EWS	PM_2.5_	MLR, MLP	Nil
8	Tudor et al. (37)^#^	India	1970–2018 annual data	Climate-policy forecasting EWS	GHG	M1: ETS,	Nil
						M2: Holt-Winters,	
						M3: TBATS,	
						M4: ARIMA,	
						M5: STS,	
						M6: NNAR,	
						M7: Naïve	
9	Sreenivasulu et al. (38)	Delhi, India	2019–2023 (4 stations)	Analytical forecasting EWS	PM_2.5_	M1: STL-CNN-BILSTM-AM,	Vellore Institute of Technology
						M2: CNN-BILSTM,	
						M3: BILSTM,	
						M4: LSTM,	
						M5: CNN	
10	Unnikrishnan et al. (39)	South India (road networks)	2022–2023	Road-network EWS	PM_2.5_, PM_10_, NO_2_, SO_2_, CO, O_3_	M1: GDM + Residual Net + Hybrid LSTM,	Nil
						M2: LSTM,	
						M3: GRU,	
						M4: EMD-CNN	
11	Visu et al. (40)	India	2015–2023 multi-city	DL-based EWS	PM_2.5_, PM_10_, CO, NO_2_, NH_3_, O_3_, NOx, SO_2_	M1: AM-CNN-OptBiLSTM (WSA),	Nil
						M2: LSTM,	
						M3: BiLSTM,	
						M4: OptBiLSTM	
12	Hossain et al. (43)^*^	Dhaka, Bangladesh	2000–2025 (31 CAMS)	AI-driven EWS (industrial shutdown advisory)	AQI (PM_2.5_, PM_10_, SO_2_, NO_2_, CO, O_3_)	M1: RF,	Nil
						M2: DT,	
						M3: GBT,	
						M4: k-NN,	
						M5: AB,	
						M6: SVM,	
						M7: LR	
13	Yadav et al. (41)	Delhi, India	2023–2024 (39 stations)	AQEWS validation	PM_2.5_	M1: GEOS-ML (Random Forest),	NSF National Center for Atmospheric Research & NASA Health and Air Quality Program
						M2: WRF-Chem (AQEWS),	
						M3: SILAM,	
						M4: DM-Chem 330m,	
						M5: DM-Chem 1.5 km,	
						M6: Global: IFS,	
						M7: GEOS-FP,	
						M8: GEFS-Aerosols	
14	Yenkikar et al. (42)	India (multi-city)	2015–2020 (forecast to 2025)	Explainable hybrid EWS	PM_2.5_, PM_10_, NO_2_, SO_2_, CO, O_3_	M1: Hybrid RF + ARIMA,	Nil
						M2: Baseline RFR	

^#^This study evaluates a macro-level environmental forecasting system projecting long-term greenhouse gas (GHG) emission trends for climate policy. While it falls under the broader continuum of environmental predictive modeling in the region, its operational objective (multi-decadal policy benchmarking) is distinct from the acute, short-term public health early warning systems (AQEWS) evaluated in this review.

^*^Hossain et al. (43) is classified as short-term based on its acute predictive output (daily industrial shutdown advisories), despite utilizing a multi-decadal dataset (2000–2025) for model training.

AQEWS, Air quality early warning system; AQI, Air quality index; AB, AdaBoost (adaptive boosting); ARIMA, Autoregressive integrated moving average; BILSTM, Bidirectional long short-term memory; BR-ANN, Bayesian regularization artificial neural network; CNN, Convolutional neural network; CTM, Chemical transport model; DT, Decision tree; 3DVAR, Three-dimensional variational; EMD-CNN, Empirical mode decomposition—Convolutional neural network; ETS, Exponential smoothing state space model; EWS, Early warning system; GBT, Gradient boosted trees; GDM, Group method of data handling; GEFS, Global ensemble forecast system; GEOS, Goddard earth observing system; GHG, Greenhouse gas; GOCART, Goddard chemistry aerosol radiation and transport model; GRU, Gated recurrent unit; IFS, Integrated forecasting system; Lasso, Least absolute shrinkage and selection operator; LightGBM, Light gradient boosting machine; LM-ANN, Levenberg–Marquardt artificial neural network; LR, Linear regression; LSTM, Long short-term memory; MAE, Mean absolute error; ML, Machine learning; MLP, Multi-layer perceptron; MLR, Multiple linear regression; MSE, Mean squared error; NN, Neural network; NNAR, Neural network autoregression; PM, Particulate matter; Residual Net, Residual network; R, Pearson correlation coefficient; R^2^, R-squared (or coefficient of determination); RF, Random forest; RFR, Random forest regressor; RMSE, Root mean square error; SCG-ANN, Scaled conjugate gradient artificial neural network; SILAM, System for integrated modeling of atmospheric composition; STL, Seasonal-trend decomposition using LOESS (local regression); STS, Structural time series; SVR, Support vector regression; TBATS, Trigonometric seasonality, Box-Cox transformation, ARMA errors, Trend, and Seasonal components; WRF-Chem, Weather research and forecasting model coupled with chemistry; XGBoost, eXtreme gradient boosting.

**Table 2:** Metric based evaluation of available early warning systems from the included studies.

S. no	References	Concentration (μg/m^3^/AQI)	Bias/mean bias/normalized mean bias	Error metrics			Fit metrics		Classification metrics
				RMSE	MAE	MSE	*R^2^*	(*R*)	Accuracy (%)
1	Oanh et al. (44)	(μg/m^3^) Monthly average: (330–400)	NA	(μg/m^3^) M1: (22.1); M2: (26.1)	NA	NA	M1: (0.87); M2: (0.76)	NA	NA
2	Jebamalar et al. (32)	NA	NA	M1: (3-4); M2: (33-34); M3: (32-33); M4: (28-29); M5: (5-6); M6: (5); M7: (4-5); M8: (4)	M1: (0.8–1.0); M2: (8.5–9.0); M3: (3.5–4.0); M4: (3.0–3.2); M5: (2.8–3.0); M6: (1.5–1.8); M7: (2.5–2.8); M8: (1.1–1.3)	NA	NA	NA	M1: (~1.0); M2: (0.972); M3: (0.971); M4: (0.973); M5: (0.986); M6: (0.990); M7: (0.975); M8: (0.997)
3	Ghude et al. (33)	Peak (μg/m^3^) (~600); M1: (~350); M2: (~170)	(μg/m^3^): Bias M1: (133); M2: (435) Mean bias M1: (−48); M2: (−150)	NA	NA	NA	NA	NA	NA
4	Jena et al. (34)	Pre WRF: (133 ± 88); M1: (135 ± 83); M2: (90 ± 36)	M1: (+1%); M2: (−32%)	NA	NA	NA	NA	NA	NA
5	Gulati et al. (35)	Observed (μg/m^3^): (0–120)	NA	(μg/m^3^): M1: (9.52); M2: (9.65); M3: (11.02); M4: (11.76)	NA	NA	M1: (0.816); M2: (0.812); M3: (0.755); M4: (0.720)	NA	NA
6	Kalita et al. (36)	(μg/m^3^) PM_10_ Gujarat: M1: (267.3); M2: (115.9); Mumbai: M1: (316.1); M2: (183.3);	(%) PM_10_ Gujarat: M1: (13.76); M2: (−57.44); Mumbai: M1: (0.35); M2: (−44.61);	(μg/m^3^) PM_10_ Gujarat: M1: (110.49); M2: (157.92); Mumbai: M1: (102.88); M2: (211.70);	NA	NA	NA	NA	NA
		Pune: M1: (181.8); M2: (95.8) PM_2.5_ Gujarat: M1: (62.7); M2: (39.8); Mumbai: M1: (62.2); M2: (47.4); Pune: M1: (59.5); M2: (38.2)	Pune: M1: (13.23); M2: (−42.14) PM_2.5_ Gujarat: M1: (34.7); M2: (−65.41); Mumbai: M1: (46.9); M2: (−59.7); Pune: M1: (41.64); M2: (−63.44)	Pune: M1: (131.43); M2: (125.11) PM_2.5_ Gujarat: M1: (47.31); M2: (73.85); Mumbai: M1: (74.10); M2: (87.45); Pune: M1: (78.46); M2: (84.74)					
7	Saiohai et al. (45)	(μg/m^3^) Daily: (15 - 35); High Pollution Episodes: (60–90); Extreme Peak (early 2019) (~110)	NA	(3h−7days data in μg/m^3^) MLR: (8.68–14.43); MLP: (9.92–15.27)	(3h−7days data in μg/m^3^) MLR: (6.00–10.39); MLP: (6.62–11.62)	NA	NA	(3h−7days) MLR: (0.86–0.52); MLP: (0.82–0.49)	NA
8	Tudor et al. (37)^#^	NA	NA	(kt of CO eq) M1: (76,108); M2: (269,141); M3: (45,626); M4: (54,134); M5: (42,551); M6: (58,231); M7: (310,503)	NA	NA	NA	NA	NA
9	Sreenivasulu et al. (38)	NA	NA	(μg/m^3^) M1: (2.93–4.61); M2: (4.26); M3: (4.26); M4: (11.2–23.0); M5: (6.5–13.2)	(μg/m^3^) M1: (2.18–3.38); M2: (4.1); M3: (5.9); M4: (10.9); M5: (5.7)	NA	M1: (0.997–0.998); M2: (0.996); M3: (0.997); M4: (0.921); M5: (0.997)	NA	NA
10	Unnikrishnan et al. (39)	(μg/Nm^3^) CO (10041–11009) NO_2_ (96–136) SO_2_ (91–142) PM10 (97–241) PM_2_.5 (99–301)	NA	(μg/Nm^3^) M1: (9.17); M2: (10.9); M3: (63.1); M4: (46.3)	NA	NA	NA	M1: (0.98); M2: (0.97); M3: (0.65); M4: (0.81)	NA
11	Visu et al. (40)	NA	NA	(μg/m^3^) M1: (0.84); M2: (1.05); M3: (1.03); M4: (1.02)	(μg/m^3^) M1: (0.53); M2: (1.23); M3: (1.16); M4: (0.90)	[(μg/m^3^) ^2^] M1: (0.72); M2: (1.11); M3: (1.07); M4: (1.04)	M1: (0.98); M2: (0.80); M3: (0.83); M4: (0.87)	NA	NA
12	Hossain et al. (43)^*^	High-risk days (≥151 AQI)	NA	(AQI index) M1: (0.127); M2: (0.221); M3: (0.466); M4: (0.24); M5: (0.146); M6: (0.415); M7: (0.41)	(AQI index) M1: (0.063); M2:(0.109); M3: (0.463); M4: (0.116); M5: (0.057); M6: (0.176); M7: (0.173)	(AQI^2^) M1: (0.023); M2: (0.061); M3: (0.217); M4: (0.064); M5: (0.033); M6: (0.176); M7: (0.173)	M1: (0.921); M2: (0.815); M3: (0.921); M4: (0.797); M5: (0.887); M6: (0.611); M7: (0.628)	NA	M1: (98.34); M2: (93.38); M3: (98.34); M4: (91.38); M5: (96.69); M6: (82.44); M7: (82.74)
13	Yadav et al. (41)	(μg/m^3^) M1: (~70–350); M2: (~50–300); M3: (~50–250); M4: (~80–250); M5: (~70–280);	(Day1 and 2 data) M1: (0.11/−0.01); M2: (−0.1/−0.12); M3: (−0.18/−0.14); M4: (−0.06/−0.06); M5: (−0.16/−0.14);	(Day1 and 2 data μg/m^3^) M1: (93.3/90.5); M2: (66.1/76.4); M3: (128.1/126.1); M4: (101.7/100.7); M5: (119.7/124.2);	NA	NA	NA	(Day1 and 2 data) M1: (0.27/0.16) M2: (0.74/0.66) M3: (0.2/0.18) M4: (0.31/0.32) M5: (0.36/0.32)	(Day1 and 2 data) M1:(100/100); M2: (94.9/94.2); M3: (86.6/88.4); M4: (95.9/96.1); M5: (87.8/88.5);
13	Yadav et al. (41)	M6: (~30–200); M7: (~40–180); M8: (~30–150)	M6: (−0.46/−0.46); M7: (−0.53/−0.62); M8: (−0.63/−0.63)	M6: (116.0/117.6); M7: (132.2/149.8); M8: (147.5/149.9)	NA	NA	NA	M6: (0.51/0.45) M7: (0.47/0.34) M8: (0.53/0.42)	M6: (65.6/64.5); M7: (51.5/30.6); M8: (18.6/15.2)
14	Yenkikar et al. (42)	NA	NA	NA	NA	(AQI^2^) M1: (508.5); M2: (524.6)	M1: (0.940); M2: (0.917)	NA	NA

^#^*T*his study evaluates a macro-level environmental forecasting system projecting long-term greenhouse gas (GHG) emission trends for climate policy. While it falls under the broader continuum of environmental predictive modeling in the region, its operational objective (multi-decadal policy benchmarking) is distinct from the acute, short-term public health early warning systems (AQEWS) evaluated in this review.

^*^Hossain et al. (43) is classified as short-term based on its acute predictive output (daily industrial shutdown advisories), despite utilizing a multi-decadal dataset (2000–2025) for model training.

AQI, Air quality index; MAE, Mean absolute error; MSE, Mean squared error; R, Pearson correlation coefficient; R^2^, R-squared (or coefficient of determination); RMSE, Root mean square error; NA, Not available.

### Assessment of bias risk

The methodological quality of the included studies was critically appraised using the PROBAST+AI tool (31), a framework specifically designed to evaluate the risk of bias and applicability of prediction models utilizing artificial intelligence or regression methods. Two trained reviewers (C.M. and S.S.) independently conducted the appraisal for each individual study. Any discrepancies between the reviewers were resolved through targeted discussion and consensus with the supervising reviewers (V.S., M.K., N.P., S.A., and S.P.). While the quality assessment informed the interpretation of model reliability and predictive accuracy, an elevated risk of bias did not serve as a criterion for excluding studies from the quantitative synthesis.

Because PROBAST+AI was originally developed primarily for prediction models in healthcare, its signaling questions were applied to AQEWS studies with an environmental forecasting interpretation. The four PROBAST+AI domains were retained: participant's/data sources, predictors, outcome, and analysis. “Participants” was interpreted as the population and observational data sources represented by the environmental monitoring datasets, including monitoring stations, geographic locations, and sampling periods. “Predictors” referred to meteorological, pollutant, satellite, emissions, temporal, and other input variables used for forecasting. “Outcome” referred to the prespecified forecast target, including pollutant concentrations, AQI categories, or related air-quality outcomes. Signaling questions concerning model development, validation, missing data, overfitting, and performance assessment were retained where applicable. Questions that were specific to clinical or healthcare contexts and had no meaningful environmental equivalent were considered not applicable rather than being inferred. No substantive modification of the underlying risk-of-bias domains was made; the adaptation was limited to interpreting the signaling questions according to the environmental forecasting context.

## Results

### Study selection and characteristics

The systematic literature search, concluded on February 10, 2026, identified 839 articles, from which 120 duplicates were removed. Primary screening of titles and abstracts was conducted independently by two reviewers, yielding 234 articles that aligned with the review objectives. Further, full-text examination of these articles deemed 14 studies eligible for inclusion for review (Figure 1). The inter-rater agreement (Cohen's kappa) between the two evaluating authors demonstrated high consistency, achieving 0.85 for title and abstract screening, and 0.90 for full-text evaluation.

The 14 included scientific articles were geographically distributed across the WHO SEARO (Figure 2), predominantly encompassing India (11 studies) (32–42), Bangladesh (one study) (43), and Thailand (two studies) (44, 45). These studies collectively covered a diverse range of environments, including highly polluted urban megacities (Delhi, Dhaka, Chennai, Bangkok), multi-city national monitoring networks (e.g., India's nationwide Central Pollution Control Board datasets), and specialized regional contexts such as dust events in Western India, seasonal haze episodes in Chiang Mai, and road network exposures in South India (Table 1). The mean observation period across the studies varied substantially, ranging from short-term operational forecasts like 72-h AQEWS validation (41) to multi-decadal horizons for climate and policy forecasting like 2001–2008 haze analysis (44), 1970–2018 greenhouse gas policy forecasting (37), 2000–2025 CAMS data in Dhaka (43). On average, the studies utilized between two and 5 years of observational or forecast data. To accommodate this broad temporal spectrum, the synthesized literature captures the region's complete predictive infrastructure. This includes systems explicitly designed for immediate public health mitigation during acute pollution episodes (operational AQEWS), as well as macro-level environmental models projecting extended emission trends, such as multi-decadal greenhouse gas policy forecasting (37). To ensure conceptual clarity and prevent conflation with acute emergency response networks, long-term policy forecasting models have been explicitly annotated within the data synthesis.

**Figure 2:**
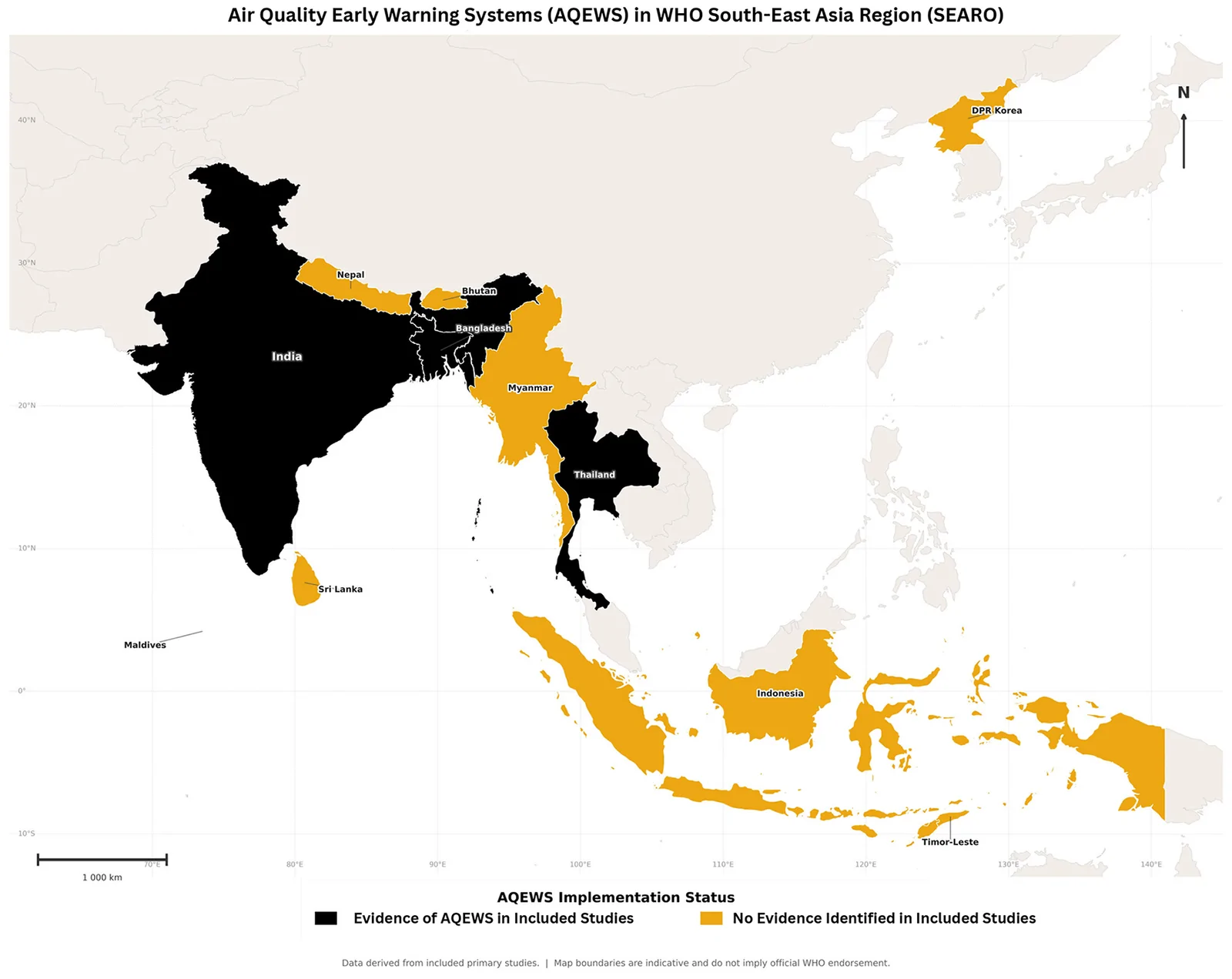
Geographical distribution of published AQEWS evidence in the WHO South-East Asia region.

### Data synthesis and model categorization

Given the substantial heterogeneity in the utilized datasets, target pollutants, modeling architectures, and evaluation metrics, direct quantitative comparison or ranking of models across the studies was precluded. Consequently, model performance was synthesized and interpreted within the individual study contexts.

The review encompassed predictive models for a wide spectrum of pollutants, including PM_2.5_, PM_10_, CO, NO_2_, SO_2_, O_3_, NO_X_, NH_3_ and greenhouse gas (GHG) emissions. PM_2.5_ was the most frequently analyzed pollutant, reflecting its dominant role in determining public health risk (32–36, 38–43, 45). Evaluated forecasting architectures ranged from chemical transport models (CTMs) enhanced by satellite data assimilation to advanced machine learning (ML) and deep learning (DL) networks. Notably, hybrid architectures incorporating spatial attention modules or statistical baselines consistently demonstrated superior spatiotemporal forecasting capabilities, achieving the highest aggregate accuracy while significantly reducing forecast bias compared to standalone models.

### Root mean square error (RMSE) estimates

Across the included literature, RMSE remained the most consistently reported performance metric. The lowest RMSE values were achieved by advanced deep learning (40) and hybrid ensemble models (32, 38), which systematically outperformed traditional regression approaches. Specifically, an STL-CNN-BiLSTM-AM model applied in Delhi (38) achieved RMSE values ranging between 2.93 and 4.61 μg/m^3^, coupled with near-perfect explanatory power (*R*^2^ = 0.997–0.998). Similarly, Visu (India, 2024) reported an RMSE of 0.84 μg/m^3^ for an AM-CNN-OptBiLSTM model, demonstrating marked superiority over standard LSTM and BiLSTM comparators (40).

Artificial Neural Network (ANN) models applied at a national scale in India achieved moderate predictive improvements, reducing the RMSE to approximately 9.5 μg/m^3^ compared to multiple linear regression (MLR) baselines of ~12 μg/m^3^ (35). Conversely, traditional regression methods such as MLR (35) and linear regression (32) consistently yielded higher RMSE values ranging from 11 to 34 μg/m^3^, underscoring their limited utility for operational early warning systems in high-pollution contexts (Table 2).

### Bias and concentration accuracy estimates

Metrics for bias and concentration accuracy were most prominently reported in studies deploying CTMs with data assimilation. Conventional WRF-Chem simulations frequently underestimated extreme PM_2.5_ episodes; during severe pollution events in Delhi (33), baseline model underestimations reached up to 435 μg/m^3^. The incorporation of satellite aerosol optical depth (AOD) and fire emission assimilation (33) reduced this forecast bias by approximately 70%, substantially improving system reliability. Jena et al. (34) in Delhi further confirmed that aerosol assimilation reduced PM_2.5_ bias by nearly 50% (~45 μg/m^3^), which enhanced categorical predictive skill for unhealthy AQI levels.

During a rare dust event in Western India (36), data assimilation reduced PM_10_ bias from between −44% and −57% to near-zero levels (~0–13%), while absolute PM_2.5_ concentrations improved by 30–38 μg/m^3^. Furthermore, Yadav (Delhi, 2025) (41) demonstrated that ensemble mean models achieved near-zero normalized mean bias and mean fractional bias, systematically outperforming individual CTMs. These findings confirm that the assimilation of satellite data and emissions inventories fundamentally positions WRF-Chem frameworks as the most reliable CTM architecture for severe pollution forecasting (Table 3).

**Table 3:** Summary of evaluated air quality early warning system models and their predictive performance.

Pollutant(s) studied	Models and algorithms evaluated	Study-specific high performing models and key metrics
**Fine Particulate Matter (PM**_**2.5**_ **)**	**CTMs** (33, 34, 36, 41)**:** WRF-Chem (with/without assimilation), SILAM, DM-Chem, GEOS-FP, IFS	**CTMs with assimilation:** WRF-Chem incorporating satellite AOD and fire emissions reduced forecast bias by ~50%−70%.
	**ML** (32, 41, 43–45)**:** RF, DT, LR, Lasso, SVR, NN, XGBoost, ANN (LM, BR, SCG)	
	**DL** (35, 38–40)**:** LSTM, BiLSTM, CNN	
	**Hybrid** (32, 38, 39, 42)**:** LightGBM+DT, STL-CNN-BiLSTM-AM, AM-CNN-OptBiLSTM, RF+ARIMA	**Hybrid deep learning:** AM-CNN-OptBiLSTM and STL-CNN-BiLSTM-AM achieved the lowest RMSE (0.84–4.61 μg/m^3^) and highest explanatory power (*R^2^* = 0.98–0.998).
**Coarse Particulate Matter (PM**_**10**_ **)**	**CTMs** (34, 36, 41)**:** WRF-Chem (with/without GOCART assimilation)	**CTMs with assimilation:** WRF-Chem with 3DVAR effectively corrected severe underestimations during dust events, reducing bias from up to −57% to near-zero levels (0–13%).
	**DL** (35, 38–40)/**Hybrid:** AM-CNN-OptBiLSTM, Residual Net + Hybrid LSTM, GRU, EMD-CNN	
	**Statistical:** Synoptic + Regression	
**Aggregate AQI and Multi-Pollutant** *(CO, NO_2_, SO_2_, O3, NH3, NOx)*	**ML** (32, 41, 43–45)**:** RF, DT, GBT, k-NN, AB, SVM, LR	**Machine learning:** Random Forest (RF) achieved peak accuracy (98.34%) and R^2^ (0.921) with the lowest MAE/MSE for categorizing high-risk industrial pollution days.
	**DL** (35, 38–40)**/Hybrid:** AM-CNN-OptBiLSTM, Residual Net + Hybrid LSTM, Hybrid RF+ARIMA	**Hybrid:** AM-CNN-OptBiLSTM consistently outperformed baselines across multi-pollutant estimations.
**Greenhouse Gases (GHG)**	**Policy-level time series** (37)**:** ETS, Holt-Winters, TBATS, ARIMA, STS, NNAR, Naive	**Structural time series (STS):** Demonstrated the lowest long-term forecast error magnitude (42,551 kt of CO eq) among evaluated baselines.

Models predicting AQI categories are treated as classification models, whereas pollutant concentration forecasting models are treated as regression models. Synthesis of the air quality forecasting architectures evaluated across the included studies, stratified by target pollutant. The table highlights the superior predictive accuracy of hybrid deep learning architectures and the critical role of data assimilation in reducing bias for chemical transport models. (CTMs, Chemical transport models; ML, Machine learning; DL, Deep learning; AQI, Air quality index; RMSE, Root mean square error; AOD, Aerosol optical depth).

### Predictive accuracy and explained variance

Predictive accuracy and explained variance (*R*^2^) were high in several machine learning (32, 43) and hybrid (38, 40, 42) model frameworks, although direct comparison across studies was limited by differences in datasets, pollutants, forecast horizons, and validation strategies. A Random Forest algorithm applied in Dhaka achieved 98.34% categorical accuracy with an *R*^2^ of 0.921, simultaneously recording the lowest Mean Absolute Error (0.063 AQI) and Mean Squared Error (0.023 AQI^2^) among all tested algorithms (43). Although this represents high reported classification performance within the evaluated dataset, overall accuracy alone is insufficient to assess the reliability of an early-warning classification system. Sensitivity, specificity, precision, F1 score, balanced accuracy, AUROC, class distribution, and confusion-matrix information were not consistently reported in the included studies. Therefore, the 98.34% accuracy should be interpreted as a study-specific performance estimate rather than evidence of exceptionally reliable early-warning capability. In particular, without information on class distribution and sensitivity to high-pollution categories, the extent to which the model successfully identified pollution episodes cannot be determined. A hybrid LightGBM combined with decision trees in Chennai achieved near-perfect accuracy (~1.0), comprehensively outperforming regression and shallow learning models across all recorded metrics (32).

Deep learning approaches applied in India, such as the STL-CNN-BiLSTM-AM (38) and AM-CNN-OptBiLSTM (40) architectures, explained more than 98% of variance in the respective study datasets. However, these findings should be interpreted in the context of study-specific datasets, forecast horizons, and validation approaches. In contrast, regression models such as MLR (35) and linear regression (43) demonstrated lower explained variance, with R^2^ values ranging from 0.68 to 0.72. Overall, these findings indicate that Random Forest and hybrid deep-learning models achieved high reported predictive performance in individual study settings; however, the strength and generalisability of these estimates depended on the underlying validation and analytical approaches (Table 3).

Importantly, high reported predictive performance did not necessarily indicate high confidence in model performance. Several hybrid and deep-learning models reported high *R*^2^ values, including values approaching 0.98–0.99, but these estimates were obtained under study-specific validation and analytical conditions. The PROBAST+AI assessment identified methodological concerns in some studies, particularly related to model analysis and validation, which reduced confidence in the generalisability of the corresponding performance estimates. Consequently, numerical performance estimates were interpreted separately from the methodological strength supporting those estimates. Models yielding higher *R*^2^ values were not considered inherently superior when the validation strategy was limited, internally based, or insufficiently described.

Across the synthesized literature, predictive performance varied according to pollutant, forecast horizon, data source, validation strategy, and operational setting. Models showing lower error within individual studies did not necessarily provide stronger evidence of generalisability, particularly when evaluation relied on internal validation or retrospective datasets. The apparent advantage of hybrid and deep-learning approaches was therefore study-specific rather than consistently demonstrated across independently validated operational AQEWS. For classification-based forecasting, these results were interpreted cautiously because overall accuracy may be strongly influenced by the distribution of pollution categories, particularly when high-pollution episodes are relatively infrequent.

### Risk of bias and methodological quality

Two independent reviewers (C.M. and S.S.) conducted the PROBAST+AI methodological quality assessment, achieving an acceptable inter-rater agreement (Cohen's kappa = 0.87) established through a pilot test of five studies. The critical appraisal revealed significant methodological variability, with an overall high risk of bias across the synthesized literature.

This high risk of bias was predominantly driven by deficiencies within the analysis domain (Domain 4) (32, 33, 35, 36, 38–40, 44). During model development, Domain 4 consistently raised high concerns due to an absence of external validation strategies, poor handling of missing data, insufficient control for algorithmic overfitting, and the inadequate reporting of analytical methods. Only a minority of studies (37, 42) demonstrated low to moderate concern in this domain due to the application of robust validation protocols.

In Domain 1 (participants and data sources), most studies presented low to moderate concern; however, high concern was noted in select studies relying on limited geographic coverage, single-sensor inputs, secondary datasets, or unclear sampling methods (32, 35, 39, 40). Studies utilizing consolidated datasets from multiple stations generally displayed low concern (33, 34, 37, 41, 42). For Domain 2 (predictors), studies were predominantly rated as low to moderate concern, though instances of high concern emerged primarily from outcome-driven predictor selection and potential time-series data leakage (35, 38). Domain 3 (outcomes) exhibited mostly low concern reflecting the uniform use of standardized PM_2.5_ or AQI outcome metrics; moderate concerns were limited to studies exhibiting calibration issues, temporal aggregation, or predictor-outcome overlap (32, 34–36, 38). The model development summary pointed out that most studies had high overall quality, while only a few studies showing low to moderate concern (37, 41, 42, 45). Overall applicability during development was generally low, but moderate concerns were seen in selected studies (32, 40, 44).

Notably, model evaluation uncovered an even higher risk of bias compared to the development phase. A significant proportion of the literature was classified as having a high risk of bias due to the lack of external validation, limited performance metrics, and a failure to assess calibration (32–35, 39, 40, 45). Only a few studies reached a low to moderate overall risk (37, 42), while some studies showed moderate risk but with relatively better validation approaches (41, 43). While applicability concerns during both development and evaluation were generally classified as low, moderate concerns persisted in several studies (32, 39, 40, 44, 45) regarding sample representativeness and dataset characteristics. Ultimately, while outcome measurement and predictor selection were generally acceptable, the frequent omission of key methodological details such as train-test splits, cross-validation strategies, or confusion matrices, severely limits the assessment of model robustness across the current evidence base (Table 4).

**Table 4:** Summary of PROBAST+AI assessment of included studies (model development and evaluation).

References	RoB					Applicability
	D1	D2	D3	D4	Overall RoB	
Oanh et al. (44)	Low -Moderate	Moderate	Low	Moderate -High	High	Moderate
Jebamalar et al. (32)	High	Moderate	Moderate	High	High	Moderate
Ghude et al. (33)	Low	Low	Low	High	High	Low
Jena et al. (34)	Low	Low	Moderate	Moderate	High	Low
Gulati et al. (35)	High	High	Moderate	High	High	Low
Kalita et al. (36)	Moderate	Moderate	Moderate	High	High	Low
Saiohai et al. (45)	Low	Low	Low	High	High	Moderate
Tudor et al. (37)	Low	Moderate	Low	Low-Moderate	Low-Moderate	Low
Sreenivasulu et al. (38)	Moderate	High	Moderate	High	High	Low
Unnikrishnan et al. (39)	High	Low	Low	High	High	Low -Moderate
Visu et al. (40)	High	Low	Low	High	High	Moderate
Hossain et al. (43)	Moderate	Low	Low	Moderate	Moderate	Moderate
Yadav et al. (41)	Low	Low	Low	Moderate	Moderate	Low
Yenkikar et al. (42)	Low	Low	Low	Low	Low-Moderate	Low

RoB, Risk of bias; D, Domain; D1, Participants and data sources; D2, Predictors; D3, Outcomes; D4, Analyses.

Importantly, reported predictive performance was interpreted alongside the methodological quality of the included studies. Several models exhibiting high predictive accuracy simultaneously presented significant methodological concerns, particularly regarding data analysis, validation, and the potential for temporal data leakage. Therefore, high *R*^2^ values, low prediction errors, and high classification accuracies were treated as localized estimates of performance within their specific study settings, rather than independent evidence of robust generalisability. Ultimately, the risk-of-bias assessment was utilized to contextualize predictive performance, rather than allowing models to be ranked solely on their reported numerical metrics.

## Discussion

This systematic review synthesizes evidence from 14 studies (32–45) evaluating the predictive performance (Table 2) of AQEWS across the WHO SEARO (Figure 2), specifically within India, Bangladesh, and Thailand. Our findings highlight a rapid methodological paradigm shift in air-quality forecasting, moving from conventional deterministic models toward advanced machine learning, deep learning, and hybrid frameworks. These advanced systems have been successfully applied across diverse environmental settings ranging from highly polluted megacities like Delhi and Dhaka to regional monitoring networks capturing seasonal haze and dust events, demonstrating a robust capacity to map and forecast critical pollutants, predominantly fine particulate matter (PM_2.5_) (32–36, 38–43, 45).

A principal finding of this review was the high reported predictive performance of several machine-learning and hybrid architectures compared with traditional statistical regression models in individual study settings (38, 40). Advanced deep-learning frameworks, such as attention-enhanced CNN-BiLSTM models, achieved near-perfect explained variance (*R*^2^ approaching 0.98–0.998) and substantially reduced the Root Mean Square Error (RMSE) (38, 40) in forecasting PM_2.5_ concentrations. Similarly, Random Forest and gradient boosting (46) algorithms demonstrated exceptional categorical accuracy for Air Quality Index (AQI) prediction, indicating potentially useful predictive performance for AQI forecasting and early-warning applications. (35). Furthermore, while standalone Chemical Transport Models (CTMs) (47) like WRF-Chem are often limited by uncertainties in emission inventories, our review found that their predictive accuracy and forecast bias improved significantly (~70% bias reduction) when assimilated with satellite-derived aerosol optical depth (AOD) and real-time meteorological data. These findings suggest that hybrid frameworks combining physical atmospheric processes with data-driven machine learning may be useful for operational forecasting, although their comparative reliability requires further independent and prospective validation (33, 34, 36, 41). Agreeing with this, external studies have also consistently highlighted the importance of AOD and spatiotemporal features in enhancing model performance (48, 49). However, contrasting evidence indicates that AOD inclusion may not universally improve prediction accuracy across all modeling contexts (50), underscoring the absolute necessity for region-specific model calibration before regional deployment.

Although reductions in RMSE and high explained variance (*R*^2^ > 0.98) (40) indicate strong predictive performance within individual study datasets, their implications for public-health applications depend on the reliability and generalisability of the underlying forecasts. Accurate prediction of pollutant concentrations may reduce uncertainty in exposure estimation and support timely risk communication; however, the included studies did not consistently evaluate whether improved statistical performance translated into better exposure assessment, health-risk stratification, or operational decision-making. Therefore, these performance estimates should be interpreted as evidence of predictive capability within the evaluated settings rather than as evidence of demonstrated public-health impact or operational effectiveness.

To accurately forecast these severe episodes, the integration of spatiotemporal (49, 51) and meteorological variables proved to be a critical determinant of model performance (52, 53). Several studies demonstrated that parameters such as boundary-layer characteristics, temperature, and wind components fundamentally shape pollution dynamics (33, 34, 36, 41). Notably, seasonal and diurnal variability consistently revealed that stagnant atmospheric conditions during winter months could entirely offset the benefits of localized emission mitigation strategies (32–36, 38–43, 45). This underscores the necessity for AQEWS that dynamically account for both human emissions and atmospheric processes. In resource-constrained regions lacking extensive ground-monitoring infrastructure, these satellite-based estimations, coupled with real-time IoT sensor networks, offer a highly scalable solution for continuous exposure assessment (54).

Despite these methodological advancements, the certainty of the current evidence is severely constrained by a predominantly high risk of bias across the evaluated literature. Our PROBAST-AI assessment identified methodological concerns, particularly in the analysis domain, including limited external validation and potential concerns regarding overfitting in some studies. Furthermore, there is substantial heterogeneity in the datasets, pollutants analyzed, and modeling approaches, which precluded direct quantitative comparability via meta-analysis (32–45). Although this review was designed to evaluate AQEWS across the WHO South-East Asia Region, 10 of the 14 included studies originated from India, whereas only two studies each were identified from Bangladesh and Thailand, with no eligible studies from several other SEARO countries. Consequently, the review shows the current geographical distribution of available evidence rather than the implementation status of AQEWS across the entire region. Additionally, the literature overwhelmingly focuses on PM_2.5_, leaving a critical knowledge gap regarding the forecasting of other highly toxic pollutants, such as NO_2_, SO_2_, and ozone. Finally, many studies relied exclusively on retrospective secondary datasets rather than testing models in real-time, operational early warning environments (32, 35, 37, 40, 45). The findings should therefore be interpreted cautiously. Although several machine-learning and hybrid deep-learning models demonstrated strong predictive performance, the heterogeneity of datasets, pollutants, forecast horizons, validation procedures, and operational settings limits direct comparison across studies. Moreover, methodological concerns identified in several studies indicates that reported performance may not always translate into reliable performance in independent or operational settings. Accordingly, model architecture alone should not be considered sufficient evidence for selecting an AQEWS for public-health decision-making.

Because AQEWS forecasting involves temporally dependent observations, potential time-series leakage is an important consideration when interpreting predictive performance. Randomly dividing observations from the same time series into training and testing datasets may result in overly optimistic performance estimates if temporally related information is shared across datasets. Greater confidence can therefore be placed in models evaluated using a genuinely future or temporally separated test period that was not available during model development. Where studies did not clearly report such temporal separation, the generalisability of their reported performance was considered uncertain.

This review has several limitations. First, the exclusion of gray literature may have resulted in underrepresentation of operational AQEWS documented primarily through government reports, institutional technical reports, programme documentation, or other non-peer-reviewed sources. Second, restriction to English-language publications may have excluded relevant evidence published in local or regional languages, particularly given the geographical diversity of the WHO South-East Asia Region. These restrictions may have contributed to publication and language bias and should be considered when interpreting the completeness and generalisability of the evidence base.

Future research must prioritize the development of multi-pollutant forecasting systems that integrate advanced machine learning with physical transport models. To improve transparency and interpretability, future studies should employ explainable artificial intelligence [such as SHAP analysis (55)] to translate complex predictive outputs into actionable policy guidelines. Most importantly, the field requires the urgent adoption of standardized methodological reporting mandating the publication of train-test splits, cross-validation strategies, and calibration metrics to ensure reproducibility. Finally, subsequent investigations must bridge the gap between environmental modeling and public health by directly assessing how the operational deployment of AQEWS mitigates population exposure and alters health trajectories during severe pollution episodes.

An important distinction emerging from this review is that reported predictive performance should not be equated with confidence in predictive performance. Several hybrid and deep-learning models achieved *R*^2^ values approaching 0.98–0.99; however, some of these studies had methodological concerns identified by the PROBAST-AI assessment, particularly regarding validation and analysis. Thus, a model reporting an *R*^2^ of 0.99 under internal or inadequately described validation cannot necessarily be considered more reliable than a model reporting an *R*^2^ of 0.90 under rigorous independent temporal validation. For AQEWS, the strength of evidence should therefore be judged not only by numerical performance but also by the appropriateness of the validation design, independence of the test data, transparency of model development, and assessment of calibration and uncertainty.

## Conclusion

Air quality early warning systems in the SEARO region are rapidly evolving through the integration of deep learning, machine learning, and data-assimilated chemical transport models. While these hybrid frameworks demonstrate high predictive performance, their immediate translation into regional public health policy is hindered by widespread methodological heterogeneity and a profound lack of rigorous external validation. Crucially, high reported accuracy should not be conflated with operational reliability unless supported by independent temporal validation and transparent calibration. Constrained by a small, geographically concentrated evidence base, the current literature restricts direct comparisons across the region. To fully harness these tools for disaster risk reduction, future research must establish multicountry validation networks, mandate standardized reporting frameworks, and prospectively evaluate whether these forecasting systems genuinely improve real-world public health preparedness and outcomes.

## Data Availability

The original contributions presented in the study are included in the article/Supplementary material, further inquiries can be directed to the corresponding author.
