# Supplementary material for: Prediction accuracy of available early warning systems for air pollution across WHO South-East Asia region: a systematic review
Source: Front Public Health. 2026 Sep 30;14:1945362. doi: 10.3389/fpubh.2026.1945362 (PMC13662169; doi:10.3389/fpubh.2026.1945362)
Supplement: Supplementary file 1 [file Table_1.docx]

**Supplementary table S1: Search strategy of databases**

| **Search number** | **Query** | **Results** (as on February 10, 2026) |
| --- | --- | --- |
| **MEDLINE (via PubMed)** | | |
| #3 | ("air pollution"[MeSH Terms] OR "air pollutants"[MeSH Terms] OR "air pollutants"[MeSH Terms] OR "particulate matter"[MeSH Terms] OR "particulate matter"[MeSH Terms]) AND ("early warning system"[Title/Abstract] OR "early warning systems"[Title/Abstract]) | 107 |
| #2 | "early warning system"[Title/Abstract] OR "early warning systems"[Title/Abstract] | 4,271 |
| #1 | "air pollution"[MeSH Terms] OR "air pollutants"[MeSH Terms] OR "air pollutants"[MeSH Terms] OR "particulate matter"[MeSH Terms] OR "particulate matter"[MeSH Terms] | 1,78,237 |
| **Embase** | | |
| #3 | #1 AND #2 | 112 |
| #2 | ('early warning system'/exp OR 'early warning signal'/exp OR ('early warning score'/exp OR 'early warning score':ti,ab,kw OR 'early warning scoring':ti,ab,kw OR 'early warning system score':ti,ab,kw OR 'physiological early warning score':ti,ab,kw)) | 8,327 |
| #1 | ('air pollution'/exp OR 'aerial pollution':ti,ab,kw OR 'aerogenic pollution':ti,ab,kw OR 'air contamination':ti,ab,kw OR 'air pollution':ti,ab,kw OR 'air pollutioning':ti,ab,kw OR 'air-borne pollution':ti,ab,kw OR 'airborne pollution':ti,ab,kw OR 'atmosphere pollution':ti,ab,kw OR 'atmospheric pollution':ti,ab,kw OR 'polluted air':ti,ab,kw OR 'polluted atmosphere':ti,ab,kw OR 'pollution, air':ti,ab,kw) OR ('air pollutant'/exp OR 'air pollutant':ti,ab,kw OR 'air pollutants':ti,ab,kw OR 'air pollutants, environmental':ti,ab,kw OR 'air pollutants, occupational':ti,ab,kw OR 'air pollutants, radioactive':ti,ab,kw OR 'pollutant, air':ti,ab,kw) OR (' PM_2·5_ exposure'/exp OR PM_2·5_ exposure':ti,ab,kw OR ' PM_2·5_ exposure':ti,ab,kw OR 'exposure to PM_2·5_':ti,ab,kw OR 'exposure to PM_2·5_':ti,ab,kw OR 'fine particle exposure':ti,ab,kw OR 'fine particulate matter exposure':ti,ab,kw OR 'particulate matter 2.5 exposure':ti,ab,kw) OR ('particulate matter exposure'/exp OR 'PM exposure (particulate matter)':ti,ab,kw OR 'PM inhalation exposure':ti,ab,kw OR 'PM pollutant exposure':ti,ab,kw OR 'exposure to particulate matter':ti,ab,kw OR 'exposure to particulates':ti,ab,kw OR 'particular matter (PM) exposure':ti,ab,kw OR 'particular matter exposure':ti,ab,kw OR 'particulate matter (PM) exposure':ti,ab,kw OR 'particulate matter exposure':ti,ab,kw OR 'particulates exposure':ti,ab,kw) | 2,71,617 |
| **Web of Science** | | |
| #3 | #1 AND #2 | 133 |
| #2 | TS=(("early warning system" OR "early warning signal" OR "early warning score" OR "early warning scoring" OR "early warning system score" OR "physiological early warning score")) | 8708 |
| #1 | TS=(("air pollution" OR "aerial pollution" OR "aerogenic pollution" OR "air contamination" OR "air pollutioning" OR "air-borne pollution" OR "airborne pollution" OR "atmosphere pollution" OR "atmospheric pollution" OR "polluted air" OR "polluted atmosphere" OR "pollution, air" OR "air pollutant" OR "air pollutants" OR "air pollutants, environmental" OR "air pollutants, occupational" OR "air pollutants, radioactive" OR "pollutant, air" OR " PM_2·5_ exposure" OR " PM_2·5_ exposure" OR "exposure to PM_2·5_" OR "exposure to PM_2·5_" OR "fine particle exposure" OR "fine particulate matter exposure" OR "particulate matter 2.5 exposure" OR "particulate matter exposure" OR "PM exposure" OR "PM inhalation exposure" OR "PM pollutant exposure" OR "exposure to particulate matter" OR "exposure to particulates" OR "particular matter exposure" OR "particulate matter (PM) exposure" OR "particulates exposure")) | 137052 |
| **Scopus** | | |
| #3 | #1 AND #2 | 452 |
| #2 | TITLE-ABS-KEY ( ( "early warning system" OR "early warning signal" OR "early warning score" OR "early warning scoring" OR "early warning system score" ) ) | 29102 |
| #1 | TITLE-ABS-KEY ( ( "air pollution" OR "aerial pollution" OR "aerogenic pollution" OR "air contamination" OR "air pollutioning" OR "air-borne pollution" OR "airborne pollution" OR "atmosphere pollution" OR "atmospheric pollution" OR "polluted air" OR "polluted atmosphere" OR "pollution, air" OR "air pollutant" OR "air pollutants" OR "air pollutants, environmental" OR "air pollutants, occupational" OR "air pollutants, radioactive" OR "pollutant, air" OR " PM_2·5_ exposure" OR " PM_2·5_ exposure" OR "exposure to PM_2·5_" OR "exposure to PM_2·5_" OR "fine particle exposure" OR "fine particulate matter exposure" OR "particulate matter 2.5 exposure" OR "particulate matter exposure" OR "PM exposure" OR "PM inhalation exposure" OR "PM pollutant exposure" OR "exposure to particulate matter" OR "exposure to particulates" OR "particular matter exposure" OR "particulate matter (PM) exposure" OR "particulates exposure" ) ) | 367177 |
| **Google scholar** | | |
| #1 | ("air pollution" OR "air pollutant" OR "particulate matter" OR "PM2.5") AND ("early warning system" OR "early warning systems" OR "early warning signal" OR "early warning score") | 35 (relevant articles in first 200 articles) |

**Supplementary table S2: PROBAST-AI Assessment of a) model development and b) model evaluation across the domains for all included studies**

**A) MODEL DEVELOPMENT**

**DOMAIN 1**: Participants and Data Sources

| **Study** | **1**·**1** | **1**·**2** | **1**·**3** | **Overall Concern** | **Key Rationale** |
| --- | --- | --- | --- | --- | --- |
| Oanh et al (2011) | Y | PY | PY | Low-Moderate | Good monitoring data; limited geography/season |
| Jebamalar et al (2019) | PY | PY | PN | High | Single sensor; no sampling framework |
| Ghude et al (2020) | Y | Y | Y | Low | High-resolution satellite + ground data; limited to Delhi/NCR and seasonal window |
| Jena et al (2021) | Y | Y | PY | Low | Real-world multi-source data; limited region/season |
| Gulati et al (2023) | Y | PY | PN | High | Single-year CPCB data; poor representativeness |
| Kalita et al (2023) | Y | PY | PN | Moderate | Event-based dataset (dust storm) |
| Saiohai et al (2023) | Y | Y | PY | Low | Urban monitoring appropriate |
| Tudor et al (2021) | Y | Y | Y | Low | Global dataset (top emitters) |
| Sreenivasulu et al (2024) | Y | PY | PY | Moderate | Limited stations |
| Unnikrishnan et al (2024) | PY | PY | PN | High | Limited geographic coverage |
| Visu et al (2024) | PY | PY | PN | High | Secondary dataset, unclear sampling |
| Hossain et al (2025) | Y | PY | PY | Moderate | Longitudinal but single-city |
| Yadav et al (2025) | Y | Y | PY | Low | Multi-station CPCB dataset |
| Yenkikar et al (2025) | Y | Y | PY | Low | Multi-year AQI dataset |

Y: Yes, N: No, PY: Probably Yes, PN: Probably No, NI: no information

1·1 Were appropriate data sources used?

1·2 Was an appropriate study design used?

1·3 Did the inclusions and exclusions of study participants result in a representative dataset?

**B. Applicability**

| **Study** | **Applicability Concern** | **Key Rationale** |
| --- | --- | --- |
| Oanh et al (2011) | Low | Population and setting aligned with intended forecasting use. |
| Jebamalar et al (2019) | Low | Dataset representative of the intended application setting. |
| Ghude et al (2020) | Low –Moderate | Model intended for haze prediction in similar terrain, but limited generalizability outside Northern Thailand-like settings. |
| Jena et al (2021) | Moderate | Single-location data may limit generalizability. |
| Gulati et al (2023) | Low | Population and intended use closely matched. |
| Kalita et al (2023) | Low | Operational data directly reflected the intended application. |
| Saiohai et al (2023) | Moderate | Urban-specific data may limit applicability to other settings. |
| Tudor et al (2021) | Low | Population and outcomes aligned with the intended forecasting objective. |
| Sreenivasulu et al (2024) | Low | Study setting directly matched the review objective. |
| Unnikrishnan et al (2024) | Low -Moderate | Real-world data were relevant, though applicability beyond similar settings may be limited. |
| Visu et al (2024) | Moderate | Dataset appropriate, but wider applicability remains uncertain. |
| Hossain et al (2025) | Moderate | Geographic specificity may limit generalizability |
| Yadav et al (2025) | Low | Real-world monitoring data directly supported the intended use |
| Yenkikar et al (2025) | Low | Dataset and outcome were consistent with the intended forecasting application. |

**DOMAIN 2**: Predictors

| **Study** | **2**·**1** | **2**·**2** | **2**·**3** | **2**·**4** | **Overall Concern** | **Key Rationale** |
| --- | --- | --- | --- | --- | --- | --- |
| Oanh et al (2011) | Y | PY | PN | Y | Moderate | Stepwise regression bias |
| Jebamalar et al (2019) | PY | PY | PN | Y | Moderate | Poor preprocessing clarity |
| Ghude et al (2020) | Y | Y | Y | Y | Low | Standard predictors (AOD, meteorology) |
| Jena et al (2021) | Y | Y | PY | Y | Low | Assimilation introduces dependency |
| Gulati et al (2023) | Y | PY | PN | Y | High | Predictor selection based on outcome |
| Kalita et al (2023) | Y | Y | PN | Y | Moderate | Predictor-outcome overlap |
| Saiohai et al (2023) | Y | PY | Y | Y | Low | Strong scientific predictors |
| Tudor et al (2021) | Y | Y | Y | Y | Moderate | Univariate predictors |
| Sreenivasulu et al (2024) | Y | Y | N | Y | High | Time-series leakage risk |
| Unnikrishnan et al (2024) | Y | PY | PY | Y | Low | Clear predictor definition |
| Visu et al (2024) | Y | PY | PY | Y | Low | Standard preprocessing |
| Hossain et al (2025) | Y | Y | PY | Y | Low | Good predictor handling |
| Yadav et al (2025) | Y | PY | Y | Y | Low | Forecast-based predictors |
| Yenkikar et al (2025) | Y | Y | Y | Y | Low | Explainable predictors |

Y: Yes, N: No, PY: Probably Yes, PN: Probably No, NI: no information

2·1 Were predictors defined and assessed in a similar way for all participants?

2·2 Was any preprocessing of predictors similar for all participants?

2·3 Were predictor assessments made without knowledge of outcome data?

2·4 Were the predictors included in the model available at the time the model was intended to be used?

**B. Applicability**

| **Study** | **Applicability Concern** | **Key Rationale** |
| --- | --- | --- |
| Oanh et al (2011) | Low | Predictors aligned with operational forecasting practice. |
| Jebamalar et al (2019) | Low | Predictors routinely available in monitoring systems |
| Ghude et al (2020) | Low | Predictors were measurable and suitable for real-time forecasting. |
| Jena et al (2021) | Low | Predictors were routinely measurable and operationally relevant. |
| Gulati et al (2023) | Low | Predictors reflected real-world forecasting inputs. |
| Kalita et al (2023) | Low | Predictors were directly applicable to operational forecasting systems. |
| Saiohai et al (2023) | Low | Predictors were widely available and broadly applicable. |
| Tudor et al (2021) | Moderate | Predictor set may not fully represent real-world forecasting conditions. |
| Sreenivasulu et al (2024) | Low | Predictors were suitable for real-time implementation. |
| Unnikrishnan et al (2024) | Low | Predictors were readily available and operationally feasible. |
| Visu et al (2024) | Low | Predictors were routinely measurable in practice |
| Hossain et al (2025) | Low | Predictors were relevant to the intended application |
| Yadav et al (2025) | Low | Predictors reflected operational forecasting requirements |
| Yenkikar et al (2025) | Low | Predictors were directly relevant to AQI forecasting. |

**DOMAIN 3**: Outcome

| **Study** | **3**·**1** | **3**·**2** | **3**·**3** | **3**·**4** | **Overall Concern** | **Key Rationale** |
| --- | --- | --- | --- | --- | --- | --- |
| Oanh et al (2011) | Y | Y | PY | Y | Low | Valid PM outcome |
| Jebamalar et al (2019) | PY | PY | PY | PY | Moderate | Sensor calibration unclear |
| Ghude et al (2020) | Y | Y | Y | Y | Low | Standard PM_2·5_ outcome |
| Jena et al (2021) | Y | Y | PN | Y | Moderate | Assimilation bias |
| Gulati et al (2023) | Y | Y | Y | PN | Moderate | Annual averaging issue |
| Kalita et al (2023) | Y | Y | PN | Y | Moderate | Outcome–predictor overlap |
| Saiohai et al (2023) | Y | Y | Y | Y | Low | Reliable measurement |
| Tudor et al (2021) | Y | Y | Y | Y | Low | Global standardized outcome |
| Sreenivasulu et al (2024) | Y | Y | N | Y | Moderate | Time-series dependency |
| Unnikrishnan et al (2024) | Y | Y | PY | Y | Low | Objective AQI |
| Visu et al (2024) | Y | Y | PY | Y | Low | Standard outcome |
| Hossain et al (2025) | Y | Y | PY | Y | Low | CAMS-based measurement |
| Yadav et al (2025) | Y | Y | Y | Y | Low | CPCB-based |
| Yenkikar et al (2025) | Y | Y | Y | Y | Low | AQI outcome |

Y: Yes, N: No, PY: Probably Yes, PN: Probably No, NI: no information

3·1 Were outcomes defined and assessed appropriately?

3·2 Were outcomes defined and assessed in a similar way for all participants?

3·3 Were outcome assessments made without use or knowledge of predictor data?

3·4 Was the time interval between predictor assessment and outcome assessment appropriate?

**B. Applicability**

| **Study** | **Applicability Concern** | **Key Rationale** |
| --- | --- | --- |
| Oanh et al (2011) | Low | Outcome directly relevant to air quality management |
| Jebamalar et al (2019) | Low | Outcome aligned with environmental health objectives. |
| Ghude et al (2020) | Low | PM_10_ prediction directly matches study objectives |
| Jena et al (2021) | Low | PM_2·5_ prediction directly aligned with intended use |
| Gulati et al (2023) | Low | PM_2·5_/AQI outcomes relevant to public health forecasting. |
| Kalita et al (2023) | Low | Forecast outcomes directly support air quality monitoring. |
| Saiohai et al (2023) | Low | PM_2·5_ outcome relevant to health risk assessment |
| Tudor et al (2021) | Low | Outcome directly matches climate policy and Paris Agreement targets |
| Sreenivasulu et al (2024) | Low | PM_2·5_ prediction directly relevant to intended application. |
| Unnikrishnan et al (2024) | Low | Outcome directly relevant despite limited reporting details. |
| Visu et al (2024) | Low | Prediction outcome appropriate for intended forecasting use. |
| Hossain et al (2025) | Low | AQI risk classification aligned with intended application. |
| Yadav et al (2025) | Low | PM_2·5_ and AQI outcomes directly support public health use. |
| Yenkikar et al (2025) | Low | AQI is directly aligned with intended prediction use |

**DOMAIN 4**: Analysis

| **Study** | **4**·**1** | **4**·**2** | **4**·**3** | **4**·**4** | **4**·**5** | **Overall Concern** | **Key Rationale** |
| --- | --- | --- | --- | --- | --- | --- | --- |
| Oanh et al (2011) | PY | PY | NI | NI | PN | High | No validation |
| Jebamalar et al (2019) | PN | PY | PN | NI | PN | High | No validation strategy |
| Ghude et al (2020) | Y | Y | PN | NI | PN | High | No missing data handling, no overfitting control |
| Jena et al (2021) | Y | Y | PY | NA | PN | Moderate | Large data but weak regularization |
| Gulati et al (2023) | PN | PN | NI | NI | PN | High | Small dataset, ANN overfitting |
| Kalita et al (2023) | PN | Y | NI | NA | NI | High | Event-based limitation |
| Saiohai et al (2023) | Y | PY | NI | NA | NI | High | Limited transparency |
| Tudor et al (2021) | Y | Y | Y | NA | Y | Low-Moderate | Strong validation |
| Sreenivasulu et al (2024) | PY | Y | PN | NA | PY | High | DL overfitting risk |
| Unnikrishnan et al (2024) | PN | PY | PN | NI | PN | High | Poor reporting |
| Visu et al (2024) | PN | PY | PN | NI | PN | High | Overfitting risk |
| Hossain et al (2025) | Y | Y | PY | NI | PY | Moderate | Cross-validation used |
| Yadav et al (2025) | Y | Y | PY | NI | PN | Moderate | Partial handling |
| Yenkikar et al (2025) | Y | Y | Y | NI | Y | Low | Strong methodology |

Y: Yes, N: No, PY: Probably Yes, PN: Probably No, NI: no information

4·1 Was there evidence that the sample size was reasonable?

4·2 Were continuous and categorical predictors handled appropriately?

4·3 Were participants with missing or censored data handled appropriately in the analysis?

4·4 If methods to address class imbalance were used, was the model or the model predictions recalibrated?

4·5 Were methods used to address potential model overfitting?

**B) MODEL EVALUATION**

**DOMAIN 1**: Participants and Data Sources

**A. Risk of Bias**

| **Study** | **1**·**1** | **1**·**2** | **1**·**3** | **Overall Risk** | **Key Rationale** |
| --- | --- | --- | --- | --- | --- |
| Oanh et al (2011) | Y | PY | PY | Low-Moderate | Seasonal and regional restriction |
| Jebamalar et al (2019) | PY | PY | PN | High | Single-site IoT data; weak sampling |
| Ghude et al (2020) | Y | Y | PY | Low | Representative operational dataset but region-specific |
| Jena et al (2021) | Y | Y | PY | Low | Real-world CPCB + satellite integration |
| Gulati et al (2023) | Y | PY | PN | High | Limited dataset; poor representativeness |
| Kalita et al (2023) | Y | PY | PN | Moderate | Event-based dataset |
| Saiohai et al (2023) | Y | Y | PY | Low | Urban monitoring appropriate |
| Tudor et al (2021) | Y | Y | Y | Low | Large global dataset |
| Sreenivasulu et al (2024) | Y | PY | PN | Moderate | Limited monitoring stations |
| Unnikrishnan et al (2024) | PY | PN | PN | High | Limited geographic coverage; unclear sampling strategy |
| Visu et al (2024) | PY | PN | PN | High | Secondary dataset; no representativeness framework |
| Hossain et al (2025) | Y | Y | PY | Low | Longitudinal multi-year dataset; good coverage but single-city |
| Yadav et al (2025) | Y | Y | PY | Low | Multi-station CPCB dataset; good representativeness |
| Yenkikar et al (2025) | Y | Y | PY | Low | Multi-year AQI dataset; acceptable representativeness |

Y: Yes, N: No, PY: Probably Yes, PN: Probably No, NI: no information

1·1 Were appropriate data sources used?

1·2 Was an appropriate study design used?

1·3 Did the inclusions and exclusions of study participants result in a representative dataset?

**B. Applicability**

| **Study** | **Applicability Concern** |
| --- | --- |
| Oanh et al (2011) | Low-Moderate |
| Jebamalar et al (2019) | Moderate |
| Ghude et al (2020) | Low |
| Jena et al (2021) | Low |
| Gulati et al (2023) | Low |
| Kalita et al (2023) | Low |
| Saiohai et al (2023) | Moderate |
| Tudor et al (2021) | Low |
| Sreenivasulu et al (2024) | Low |
| Unnikrishnan et al (2024) | Low-Moderate |
| Visu et al (2024) | Moderate |
| Hossain et al (2025) | Low |
| Yadav et al (2025) | Low |
| Yenkikar et al (2025) | Low |

**DOMAIN 2**: Predictors

**A. Risk of Bias**

| **Study** | **2**·**1** | **2**·**2** | **2**·**3** | **2**·**4** | **Overall Risk** | **Key Rationale** |
| --- | --- | --- | --- | --- | --- | --- |
| Oanh et al (2011) | Y | PY | PN | Y | Moderate | Stepwise regression introduces bias |
| Jebamalar et al (2019) | PY | PY | PN | Y | Moderate | Limited preprocessing transparency |
| Ghude et al (2020) | Y | Y | Y | Y | Low | Standardized predictors; consistent preprocessing |
| Jena et al (2021) | Y | Y | PY | Y | Low | Well-defined predictors; minor dependency risk |
| Gulati et al (2023) | Y | PY | PN | Y | High | Outcome-driven predictor selection; weak preprocessing |
| Kalita et al (2023) | Y | Y | PN | Y | Moderate | Predictor-outcome overlap |
| Saiohai et al (2023) | Y | PY | Y | Y | Low | Scientifically valid predictors |
| Tudor et al (2021) | Y | Y | Y | Y | Moderate | Limited predictor diversity |
| Sreenivasulu et al (2024) | Y | Y | N | Y | High | Time-series leakage risk |
| Unnikrishnan et al (2024) | Y | PY | PY | Y | Low | Predictors clearly defined; acceptable preprocessing |
| Visu et al (2024) | Y | PY | PY | Y | Low | Standard predictors; moderate preprocessing detail |
| Hossain et al (2025) | Y | PY | Y | Y | Low | Good predictor handling; real-world inputs |
| Yadav et al (2025) | Y | PY | Y | Y | Low | Forecast-based predictors; appropriate |
| Yenkikar et al (2025) | Y | Y | Y | Y | Low | Strong preprocessing; explainability included |

Y: Yes, N: No, PY: Probably Yes, PN: Probably No, NI: no information

2·1 Were predictors defined and assessed in a similar way for all participants?

2·2 Was any preprocessing of predictors similar for all participants?

2·3 Were predictor assessments made without knowledge of outcome data?

2·4 Were the predictors included in the model available at the time the model was intended to be used?

**B. Applicability**

| **Study** | **Applicability Concern** |
| --- | --- |
| Oanh et al (2011) | Low |
| Jebamalar et al (2019) | Low |
| Ghude et al (2020) | Low |
| Jena et al (2021) | Low |
| Gulati et al (2023) | Low |
| Kalita et al (2023) | Low |
| Saiohai et al (2023) | Low |
| Tudor et al (2021) | Moderate |
| Sreenivasulu et al (2024) | Low |
| Unnikrishnan et al (2024) | Low |
| Visu et al (2024) | Low |
| Hossain et al (2025) | Low |
| Yadav et al (2025) | Low |
| Yenkikar et al (2025) | Low |

**DOMAIN 3**: Outcome

**A. Risk of Bias**

| **Study** | **3**·**1** | **3**·**2** | **3**·**3** | **3**·**4** | **Overall Risk** | **Key Rationale** |
| --- | --- | --- | --- | --- | --- | --- |
| Oanh et al (2011) | Y | Y | PY | Y | Low | Valid environmental outcome |
| Jebamalar et al (2019) | PY | PY | PY | PY | Moderate | Sensor validation unclear |
| Ghude et al (2020) | Y | Y | Y | Y | Low | Standard PM_2·5_ outcome; well-defined |
| Jena et al (2021) | Y | Y | PN | Y | Moderate | Assimilation introduces bias |
| Gulati et al (2023) | Y | Y | Y | PN | Moderate | Annual averaging reduces temporal validity |
| Kalita et al (2023) | Y | Y | PN | Y | Moderate | Outcome–predictor overlap |
| Saiohai et al (2023) | Y | Y | Y | Y | Low | Reliable outcome measurement |
| Tudor et al (2021) | Y | Y | Y | Y | Low | Standardized global outcome |
| Sreenivasulu et al (2024) | Y | Y | N | Y | Moderate | Time-series dependency |
| Unnikrishnan et al (2024) | Y | Y | PY | Y | Low | AQI/pollutant outcomes clearly defined |
| Visu et al (2024) | Y | Y | PY | Y | Low | Standard outcome; objective measurement |
| Hossain et al (2025) | Y | Y | PY | Y | Low | CAMS-based monitoring; reliable |
| Yadav et al (2025) | Y | Y | Y | Y | Low | CPCB-based outcome; appropriate |
| Yenkikar et al (2025) | Y | Y | Y | Y | Low | AQI outcome; standardized |

Y: Yes, N: No, PY: Probably Yes, PN: Probably No, NI: no information

3·1 Were outcomes defined and assessed appropriately?

3·2 Were outcomes defined and assessed in a similar way for all participants?

3·3 Were outcome assessments made without use or knowledge of predictor data?

3·4 Was the time interval between predictor assessment and outcome assessment appropriate?

**B. Applicability**

| **Study** | **Applicability Concern** |
| --- | --- |
| Oanh et al (2011) | Low |
| Jebamalar et al (2019) | Low |
| Ghude et al (2020) | Low |
| Jena et al (2021) | Low |
| Gulati et al (2023) | Low |
| Kalita et al (2023) | Low |
| Saiohai et al (2023) | Low |
| Tudor et al (2021) | Low |
| Sreenivasulu et al (2024) | Low |
| Unnikrishnan et al (2024) | Low |
| Visu et al (2024) | Low |
| Hossain et al (2025) | Low |
| Yadav et al (2025) | Low |
| Yenkikar et al (2025) | Low |

**DOMAIN 4**: Analysis

**Risk of Bias**

| **Study** | **4**·**1** | **4**·**2** | **4**·**3** | **4**·**4** | **4**·**5** | **4**·**6** | **4**·**7** | **Overall Risk** | **Key Rationale** |
| --- | --- | --- | --- | --- | --- | --- | --- | --- | --- |
| Oanh et al (2011) | Y | PY | NI | NA | PN | N | PY | Moderate-High | External validation but no calibration |
| Jebamalar et al (2019) | PN | PN | PN | NA | PN | N | PN | High | No validation; high overfitting risk |
| Ghude et al (2020) | PN | Y | PN | NA | PN | NI | PY | High | No validation; no calibration |
| Jena et al (2021) | PN | Y | PY | NA | PN | NA | Y | High | No external validation |
| Gulati et al (2023) | PN | PN | NI | NA | PN | PN | PY | High | Weak validation; limited metrics |
| Kalita et al (2023) | PN | PN | NI | NA | NI | NA | PY | High | Event-based; weak robustness |
| Saiohai et al (2023) | Y | Y | PN | NA | NI | NA | PY | High | No external validation |
| Tudor et al (2021) | Y | Y | Y | NA | Y | Y | Y | Low-Moderate | Strong internal validation |
| Sreenivasulu et al (2024) | PY | Y | PN | NA | PY | PN | PY | High | DL overfitting + no external validation |
| Unnikrishnan et al (2024) | PN | PN | PN | NI | PN | PN | PY | High | Poor validation reporting |
| Visu et al (2024) | PN | PN | PN | NI | PN | PN | PY | High | Limited metrics; overfitting risk |
| Hossain et al (2025) | Y | Y | PY | NI | Y | PN | PY | Moderate | Cross-validation; no external validation |
| Yadav et al (2025) | Y | Y | PY | NI | Y | PN | PY | Moderate | Adequate validation; no calibration |
| Yenkikar et al (2025) | Y | Y | Y | NI | Y | Y | PY | Low-Moderate | Strong validation; minor gaps |

Y: Yes, N: No, PY: Probably Yes, PN: Probably No, NI: no information

4·1 Was model evaluation based on only apparent performance avoided?

4·2 Was there evidence that the sample size was reasonable?

4·3 Were participants with missing or censored data handled appropriately in the analysis?

4·4 If methods to address class imbalance were used, was the evaluation done in a dataset without correction for imbalance?

4·5 If data splitting was done to create training and test datasets, was there evidence that data leakage was avoided?

4·6 If resampling methods were used to evaluate model performance, were all model development steps replicated in the resampling process?

4·7 Was the predictive performance of the model evaluated appropriately—for example, calibration, discrimination, and net benefit?
